# Digital Phenotyping for Monitoring Mental Disorders: Systematic Review

**DOI:** 10.2196/46778

**Published:** 2023-12-13

**Authors:** Pasquale Bufano, Marco Laurino, Sara Said, Alessandro Tognetti, Danilo Menicucci

**Affiliations:** 1 Institute of Clinical Physiology National Research Council Pisa Italy; 2 Department of Surgical, Medical and Molecular Pathology and Critical Care Medicine University of Pisa Pisa Italy; 3 Department of Information Engineering University of Pisa Pisa Italy

**Keywords:** digital phenotyping, mobile, mental health, smartphone, mobile sensing, passive sensing, active sensing, digital phenotype, digital biomarker, mobile phone

## Abstract

**Background:**

The COVID-19 pandemic has increased the impact and spread of mental illness and made health services difficult to access; therefore, there is a need for remote, pervasive forms of mental health monitoring. Digital phenotyping is a new approach that uses measures extracted from spontaneous interactions with smartphones (eg, screen touches or movements) or other digital devices as markers of mental status.

**Objective:**

This review aimed to evaluate the feasibility of using digital phenotyping for predicting relapse or exacerbation of symptoms in patients with mental disorders through a systematic review of the scientific literature.

**Methods:**

Our research was carried out using 2 bibliographic databases (PubMed and Scopus) by searching articles published up to January 2023. By following the PRISMA (Preferred Reporting Items for Systematic Review and Meta-Analysis) guidelines, we started from an initial pool of 1150 scientific papers and screened and extracted a final sample of 29 papers, including studies concerning clinical populations in the field of mental health, which were aimed at predicting relapse or exacerbation of symptoms. The systematic review has been registered on the web registry *Open Science Framework*.

**Results:**

We divided the results into 4 groups according to mental disorder: schizophrenia (9/29, 31%), mood disorders (15/29, 52%), anxiety disorders (4/29, 14%), and substance use disorder (1/29, 3%). The results for the first 3 groups showed that several features (ie, mobility, location, phone use, call log, heart rate, sleep, head movements, facial and vocal characteristics, sociability, social rhythms, conversations, number of steps, screen on or screen off status, SMS text message logs, peripheral skin temperature, electrodermal activity, light exposure, and physical activity), extracted from data collected via the smartphone and wearable wristbands, can be used to create digital phenotypes that could support gold-standard assessment and could be used to predict relapse or symptom exacerbations.

**Conclusions:**

Thus, as the data were consistent for almost all the mental disorders considered (mood disorders, anxiety disorders, and schizophrenia), the feasibility of this approach was confirmed. In the future, a new model of health care management using digital devices should be integrated with the digital phenotyping approach and tailored mobile interventions (managing crises during relapse or exacerbation).

## Introduction

### Background

Two years of the pandemic increased the impact and spread of mental illness on people’s lives, and the difficulty in accessing health services during this period highlighted the need for remote, pervasive, and early forms of monitoring the mental health status. The World Health Organization, in a report published in March 2022, pointed out that the prevalence of mental disorders, already strongly present in the general population before the pandemic, increased dramatically in 2020; estimates showed a 27.6% increase in cases of major depressive disorder and a 25.6% increase in cases of anxiety disorder [1]. In addition, the pandemic has affected health services, and a 3-phase survey conducted by the World Health Organization in 69 different countries showed that most countries (92%) experienced an interruption of at least one essential health care service, pointing out that on average, approximately half (45%) of the services were interrupted [2]. It is in this context that the need for medicine to follow new directions, including in the field of mental health, has emerged even stronger than before: a direction toward remote monitoring tools that go beyond the direct observation of behavior and clinical assessment of the emotional state. These new tools should lead to so-called digital phenotyping, in that they would use as markers of mental state the measures that can be extracted from spontaneous interaction with digital technological devices, foremost among them the smartphone [3].

**Figure 1:**
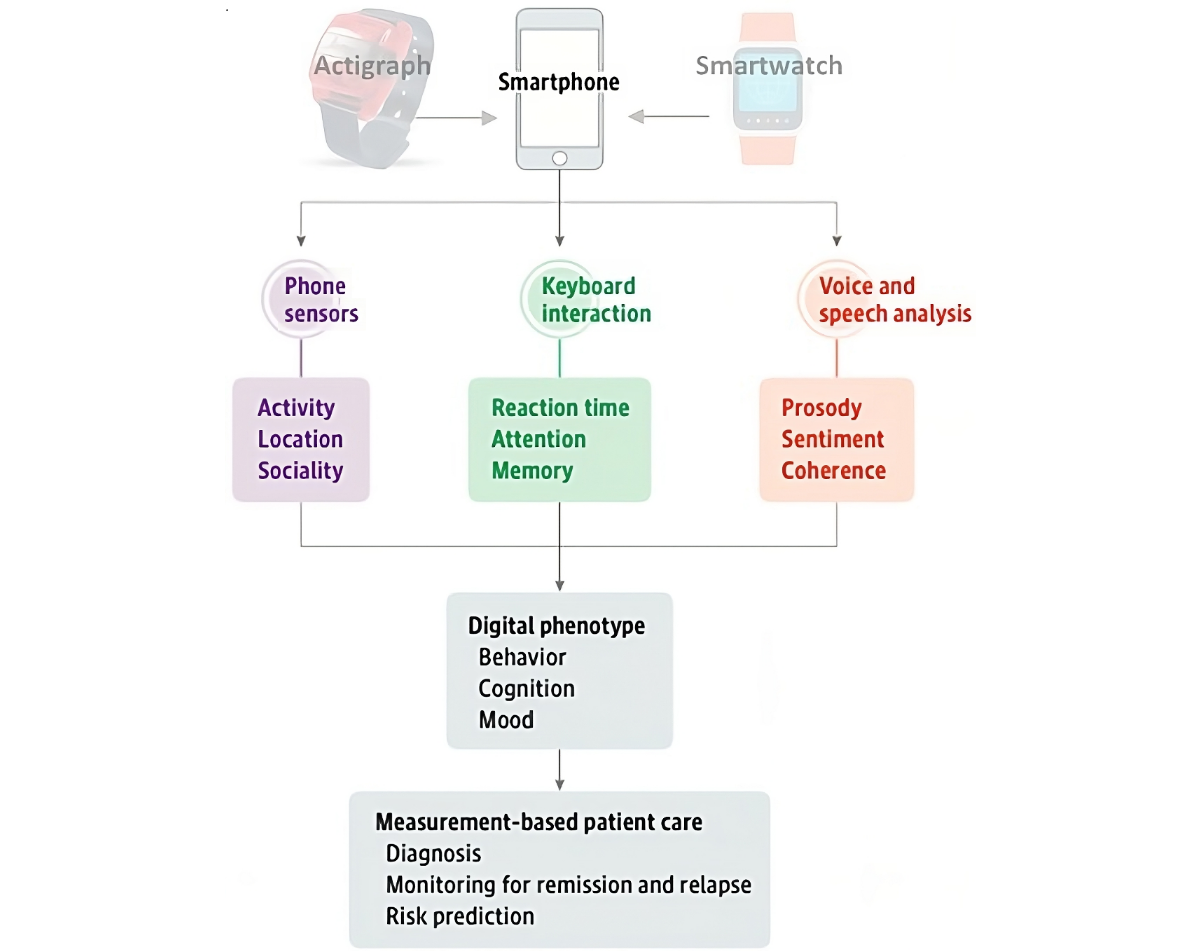
Digital phenotyping model: through the smartphone, using sensors (phone sensors), keyboard interactions (keyboard interaction), and voice and speech analysis (voice and speech analysis), it becomes possible to construct a “digital phenotype” (digital phenotype) for each participant to carve out and build the patient care process (measurement-based patient care; edited from the study by Insel [3]).

Digital phenotyping is an innovative approach concerning the extraction and analysis of information about behavior, cognition, and mood through digital tools that every person nowadays possesses. In particular, from smartphones, it is possible to passively record a large amount of information from sensors (eg, accelerometer, gyroscopes, and GPS antennas), touch screen interactions, and voice or speech analysis; other devices, such as smartwatches or ActiGraph devices, can also be used to acquire passive data, but these are still, in most cases, processed through an app installed on the smartphone. All this information can then be analyzed using big data processing methods, such as deep learning and machine learning algorithms, aimed at classifying mental health status or predicting symptomatic worsening [3] (Figure 1).

At this initial stage of validation of digital phenotyping approaches, 2 types of data are typically acquired: the first one, called passive data, comes from passive sensing without any patient intervention and the second one is called active data because the data are collected with the involvement of the patient who fills out self-report questionnaires about their mental state. Passive data can be used for digital phenotyping after appropriate validation with active data that are considered the gold standard for diagnosis, evaluation, and prevention.

This digital phenotyping approach, based on the use of validated passive data, could solve some of the limitations of current psychiatry related to the sparsity and shortness of patient assessments and the lack of objectivity and reliability in diagnosis [3-5].

Indeed, this approach could provide greater objectivity as it is based on behavioral data and could offer pervasive monitoring within populations as 3.5 billion people worldwide own smartphones [6].

### Objectives

As the digital phenotyping approach is emerging and experimental studies on selected populations with different mental health issues are rapidly accumulating, a unified picture of the effectiveness of this approach for remote monitoring of symptoms in these patients is needed. For this purpose, we carried out a systematic review concerning the use of digital phenotyping (with data acquired at least through the smartphone, both to limit the range of research and because it is the most widely used digital instrument) on patients with mental disorders for individual characterization over time and prediction of relapses or exacerbations.

## Methods

### Study Design and Search Strategy

We conducted this systematic review following the PRISMA (Preferred Reporting Items for Systematic Reviews and Meta-Analyses) guidelines [7,8] (The PRISMA checklist and PRISMA abstract checklist have been included in Multimedia Appendix 1 and Multimedia Appendix 2, respectively). The retrieval process consisted of 3 phases.

#### Preliminary Research and Definition of Keywords

During the first phase, which started in October 2022, we carried out preliminary research in the absence of specific keywords based on the seminal paper of Insel [3] to define the search criteria and identify the specific keywords to be used. On the basis of our research question, this preliminary search led us to define specific keywords (Textbox 1) related to “digital phenotyping” for inclusion in search engines to conduct systematic research.

**List of selected keywords.**

- Digital phenotyping
- Digital phenotype
- Digital biomarker
- Digital footprint
- Mobile sensing
- Passive sensing
- Smartphone

#### Systematic Search and the Definition of PICOS

The second phase, carried out in January 2023, consisted of a systematic search among titles, abstracts, and keywords of scientific papers using the electronic databases PubMed and Scopus based on the selected keywords properly combined through the Boolean operators “AND” and “OR”; no temporal restriction was set, so all articles published up to January 2023 were included in the systematic search. The search strategy, including all keywords used and the number of studies found from each database, is analytically reported in Table 1.

The final selection of papers for inclusion was carried out according to the Population, Intervention, Comparison, Outcomes, and Study Design (PICOS) worksheet [7,8], as summarized in Table 2.

**Table 1:** Search strategy.

Database	Search query	Items founded
PubMed	(“smartphone”[Title/Abstract]) AND ((“digital phenotyping”[Title/Abstract]) OR (“digital phenotype”[Title/Abstract]) OR (“digital biomarker”[Title/Abstract]) OR (“digital footprint”[Title/Abstract]) OR (“mobile sensing”[Title/Abstract]) OR (“passive sensing”[Title/Abstract]))	226
Scopus	(“smartphone”[Title/Abstract/Keywords]) AND ((“digital phenotyping”[Title/Abstract/Keywords]) OR (“digital phenotype”[Title/Abstract/Keywords]) OR (“digital biomarker”[Title/Abstract/Keywords]) OR (“digital footprint”[Title/Abstract/Keywords]) OR (“mobile sensing”[Title/Abstract/Keywords]) OR (“passive sensing”[Title/Abstract/Keywords]))	926

**Table 2:** Population, Intervention, Comparison, Outcomes, and Study Design (PICOS) worksheet.

Parameters	Inclusion criteria	Exclusion criteria
Participants	Clinical population	Healthy participants
Interventions	Mental health	Not concerning mental health
Comparisons	Control group	Theoretical study
Outcomes	Prediction or exacerbation of symptoms	Assessment-only studies
Study design	Original studies in English or Italian	Review, narrative review, systematic review, meta-analysis, and conference paper

#### Application of PICOS Study Design Exclusion Criteria

The final phase consisted of a first step in which, following the Study Design section of PICOS criteria, we excluded all reviews, both narrative and systematic reviews, meta-analyses, and conference papers, to substantially reduce the number of included studies.

#### Title and Abstract Selection

After reading the titles and abstracts, we excluded all studies that did not address the research question.

#### Full-Text Selection According to PICOS Criteria

Finally, we included in the systematic review only those papers aimed at the clinical population and related to the prediction of relapse or exacerbation of symptoms in patients with mental disorders according to the previously defined PICOS criteria. The included papers were read thoroughly to obtain the data of interest. At the end of the process, we registered the systematic review on the web registry *Open Science Framework* (registration link is findable by typing the title of the paper into the web registry search engine).

### Synthesis Method

The included papers were clustered according to mental disorder and, for each study, the following information was extracted:

Study=name of authors, year; country (region)=where the study took place; data=when the study took place; symptoms investigated=which data were collected; assessment technology=which tool was used to collect data; sample=sample size; data collection time=for how long and how often data were collected; statistics=main statistical results obtained; and synthesis of main results=narrative summary of results.

### Study Risk of Bias Assessment

We assessed the risk of bias for each study in Multimedia Appendix 3 by compiling the AXIS tool [9].

## Results

### Flow Diagram

**Figure 2:**
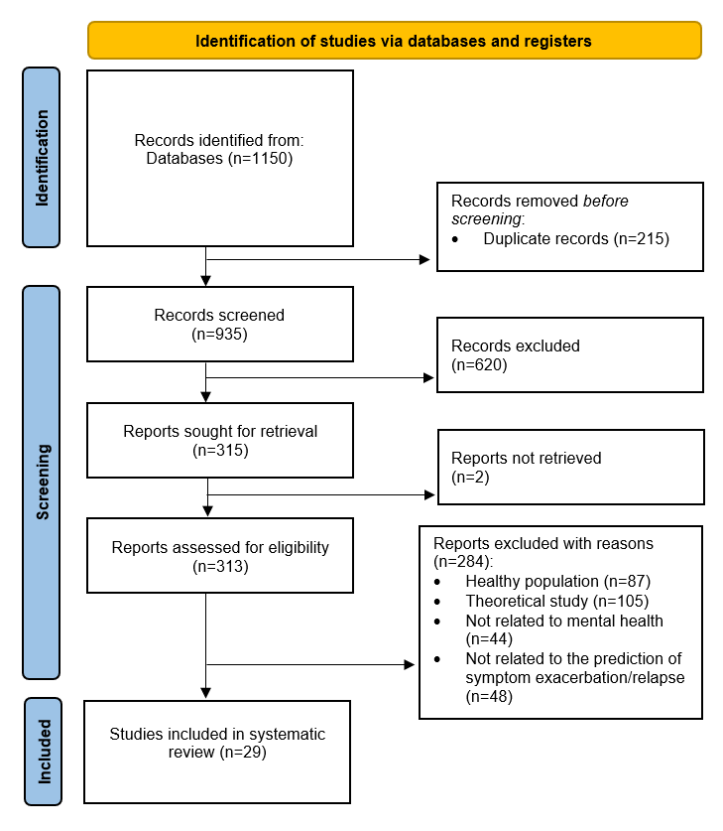
Flow diagram.

Figure 2 shows all phases of the systematic review process. The research carried out on PubMed and Scopus yielded 226 and 926 studies, respectively. These 1150 papers were merged in a nonredundant database and 935 papers remained. Then, by eliminating all studies unrelated to our research question, the number of studies was reduced to 315, of which 2 were not retrieved. Finally, by applying all the PICOS criteria, we obtained 29 studies to be included in the systematic review.

### Study Selection and Characteristics

Two independent reviewers (PB and SS) checked the pool of 935 abstracts collected from PubMed and Scopus search engine outputs, and any disagreement was discussed with author DM as the arbitrator. Titles and abstracts were screened, and 620 records were removed according to the PICOS criteria related to the study design (excluding all types of reviews, meta-analyses, and conference papers) and irrelevance to the research question. The remaining 313 papers were checked for eligibility according to the remaining PICOS criteria and all theoretical studies (n=105, 33.5%), all studies regarding a healthy population (n=87, 27.8%), all studies unrelated to mental health (n=44, 14%), and finally, all studies unrelated to the prediction of symptom exacerbation or relapse (n=48, 15.3%) were excluded. Finally, 29 articles were included, as summarized in Tables 3-6, which describe the main information of each study, divided according to disorder.

**Table 3:** Schizophrenia: retrieved studies and their main outcomes.

Study	Country (region)	Data	Psychiatric disorder	Symptoms investigated	Assessment technology	Sample, n	Data collection time	Statistics	Synthesis of main results
Abbas et al [10], 2021	New York, United States	2021	Schizophrenia	Euclidean distance of head movements frame by frame	Passive (smartphone): front-facing camera of the smartphone	Schizophrenia: 18 Controls: 9	Period of collection: 2 weeks Frequency of collection: once a day	Rate of head movement with and without schizophrenia (1.48 mm/frame vs 2.50 mm/frame; *P*=.01) Head movement predictor of schizophrenia diagnosis (*P*=.02) Head movement and negative symptoms (t=−2.245; *P*=.04)	Smartphone-based remote assessments were able to capture meaningful visual behaviors for objective measurement of head movements of patients with schizophrenia, demonstrating that it is possible to quantify the severity of their symptoms, particularly negative symptoms, by head movements.
Abbas et al [11], 2022	New York, United States	2019	Schizophrenia	Facial and vocal characteristics, including facial expressivity, vocal acoustics, and speech prevalence	Passive (smartphone): front-facing smartphone camera and microphone Active: PANSS^a^	20	Period of collection: 14 days Frequency of collection: passive (continuously) and active (twice)	Significant correlations between vocal markers and N total were fundamental frequency mean (r=−0.64; adjusted *P*=.02), vocal jitter (r=0.56; adjusted *P*=.02), and harmonics to noise ratio (r=−0.61; adjusted *P*=.02). Significant correlations between free behavior in response to images and N total: fundamental frequency mean (r=−0.61; adjusted *P*=.04), harmonics to noise ratio (r=−0.58; adjusted *P*=.03), and speech prevalence (r=−0.57; adjusted *P*=.03).	Facial and vocal measurements collected remotely in patients with schizophrenia via smartphones in response to automated task prompts demonstrated accuracy as markers of schizophrenia symptom severity.
Adler et al [12], 2020	United States	2020	Schizophrenia	Environmental data, sleep, SMS text messages, location, calls, app use, screen interaction	Passive (smartphone): app CrossCheck Active: EMA^b^	60	Period of collection: 12 months Frequency of collection: passive (continuously) and active (every 2-3 days)	Model sensitivity: 0.25 (IQR 0.15-1.00) Model specificity: 0.88 (IQR 0.14-0.96) A median 108% increase in behavioral anomalies near relapse	Using digital tools in mental health, it is possible to predict relapse in patients with schizophrenia.
Barnett et al [13], 2018	United States	2018	Schizophrenia	Mobility, sociality, mood	Passive (smartphone): GPS, accelerometer, anonymous call and SMS text message logs, screen on/off time, phone charge status Active: biweekly surveys	17	Period of collection: 3 months Frequency of collection: passive (continuously) and active (every 2 weeks)	The rate of behavioral abnormalities detected in the 2 weeks before relapse was 71% higher than during other periods.	Results showed that, with proper supporting instrumentation, smartphones can be used as research tools within mental health.
Henson et al [14], 2020	Boston, MA, United States	2020	Schizophrenia	Sociality, mobility, sleep, anxiety and depressive symptoms, psychotic symptoms	Passive (smartphone): GPS, accelerometer, screen on/off and call/SMS logs Active: PHQ-9^c^, GAD-7^d^	92	Period of collection: 3 months Frequency of collection: passive (continuously) and active (10 times in 3 months)	Clinical population: passive data features and symptoms—Spearman ρ ranged from −0.23 to −0.30, *P*<.001. Healthy controls: passive data features and symptoms—Spearman ρ ranged from 0.20 to 0.44, *P*<.05	The results suggest that digital phenotyping in schizophrenia may offer clinically relevant information for understanding how daily routines influence symptoms.
Henson et al [15], 2021	Boston, MA, United States	2021	Schizophrenia	Mobility, sociality, sleep, cognition	Passive (smartphone): GPS, calls, messages, accelerometer, screen use Active: PHQ-9, GAD-7, PANSS, CGI (clinical global impression)	Schizophrenia=83 Controls=43	Period of collection: 2 years Frequency of collection: passive (twice each day or 5 times each week) and active (every 1-3 months)	Sensitivity=89% and specificity=75% in predicting symptom relapse	The model used confirmed the potential and clinical utility of longitudinal collection of symptomatologic and behavioral data.
Ranjan et al [16], 2022	Boston, United States	2019-2021	Schizophrenia	Mood, sleep, and psychosis symptoms	Passive (smartphone): sensors (GPS, accelerometer, screen time, call and text logs) Active: PANSS and EMA	86	Period of collection: 21 months Frequency of collection: passive (continuously) and active (5 clinical visits and undefined amount of survey completion)	Correlation between gold standard and app-based self-report symptom (*r*=0.8, *P*=10^−11^ for mood and *r*=0.78, *P*=10^−12^ for anxiety)	The intraindividual symptom correlations and the stratification of symptoms by home time highlight the utility of digital phenotyping methods as a diagnostic tool, as well as the potential for personalized psychiatric treatment based on these data.
Strauss et al [17], 2022	United States	2022	Schizophrenia	Negative psychosis symptoms	Passive (wearables through smartphone app): accelerometry Passive (smartphone): accelerometry Active: SCID-5^e^, BNSS^f^, PANSS, and LOF^g^	Schizophrenia=50 Control=70	Period of collection: 6 days Frequency of collection: passive (continuously) and active (8 daily surveys)	Group differences in accelerometry: Phone ACL^h^: Mean: F1, 118=17.75, *P*<.001 SD: F1, 118=23.64, *P*<.001 Band ACL: Mean: F1, 64=.06, *P*=.8 SD: F1, 64=.60, *P*=.44 Activity index: F1, 64=0.47, *P*=.5 Convergent validity: ACL band mean and BNSS anhedonia=−0.45, *P*<.05 BNSS avolition=−0.41, *P*<.05 BNSS blunted affect=−0.4, *P*<.05 BNSS alogia=−0.4, *P*<.05	Accelerometry is a valid objective measure of negative symptoms that may complement traditional approaches to assess the construct using clinical rating scales.
Wang et al [18], 2016	Heidelberg, Germany	2016	Schizophrenia	Location, sleep, sociality, smartphone use, and environmental conditions	Passive (smartphone): CrossCheck App Active: EMA	21	Period of collection: 12 months Frequency of collection: passive (continuously) and active (3 times a week)	Mean prediction model error of 7.6% of the score range	We show that by leveraging knowledge from a population with schizophrenia, it is possible to train accurate personalized models that require less individual-specific data to rapidly adapt to new users.

^a^PANSS: Positive and Negative Syndrome Scale.

^b^EMA: Ecological Momentary Assessment

^c^PHQ-9: Patient Health Questionnaire-9.

^d^GAD-7: Generalized Anxiety Disorder-7.

^e^SCID-5: Structured Clinical Interview for Diagnostic and Statistical Manual of Mental Disorders, Fifth Edition.

^f^BNSS: Brief Negative Symptom Scale.

^g^LOF: Level of Functioning Scale.

^h^ACL: Accelerometry.

**Table 4:** Mood disorders: retrieved studies and their main outcomes.

Study			Country (region)		Data		Psychiatric disorder		Symptoms investigated		Assessment technology		Sample, n		Data collection time		Statistics		Synthesis of main results
**Bipolar disorder**																			
	Busk et al [19], 2020	Denmark		2020		Bipolar disorder		Activity, alcohol use, anxiety, irritability, cognitive difficulties, medication, mood, sleep, and stress		Active: self-report questionnaire on mood, HDRS (Hamilton Depression Rating Scale), YMRS (Young Mania Rating Scale)		84		Period of collection: 9 months Frequency of collection: active (5 times, expert ratings and daily, self-ratings)		Hierarchical Bayesian regression model History of 4 days of self-assessment: R2=0.51; RMSE^a^=0.32		The proposed method can predict mood for up to 7 days with low error compared with classical machine learning methods.	
	Ebner-Priemer et al [20], 2020	Karlsruhe, Germany		2020		Bipolar disorder		Sleep, activity, and sociality		Passive (smartphone): frequency and length of calls, screen illumination, speed of transmitted and received data, distance traveled, frequency of different classes of activity, speed of movement and number of steps Active: expert ratings and self-ratings		29		Period of collection: 12 months Frequency of collection: passive (continuously) and active (biweekly, expert ratings and daily, self-ratings)		Day-to-day association: mania status and activity (β=.123; 95% CI [0.075 to 0.170]). mania and sleep (β=−.098 95% CI [−0.157 to −0.040])		Results showed that shorter sleep duration and increased activity were associated with higher levels of mania, whereas higher than average activity correlated with lower levels of depression.	
	Faurholt-Jepsen et al [21], 2021	Copenhagen, Denmark		2021		Bipolar disorder		Movement (place, stops, moves), location entropy, routine (average distances for each day)		Passive (smartphone): GPS, Wi-Fi, mobile repeater signals Active: daily self-report of mood		bipolar=46 controls=31		Period of collection: 9 months Frequency of collection: passive (continuously) and active (daily)		Location entropy: BD^b^ vs HC^c^ (β=−.14; 95% CI=−0.24 to −0.034; *P*=.009)		This study shows how alterations in location data, reflecting patterns of mobility, may prove to be a promising measure of illness and disease fluctuations in patients with bipolar disorder, and can be used to monitor the effects of treatments.	
	Tseng et al [22], 2022	Taiwan		2020-2021		Bipolar disorder		Sleep and emotional status		Passive (smartphone): GPS data Active: YMRS, HAMD, ASRM^d^, and DASS^e^-21; daily mood, walking time, and bed time		159		Period of collection: 13 months Frequency of collection: passive (continuously) and active (daily)		Associations Daily mood: Week-to-week: Movement (r=0.213; *P*<.001) Month-to-month Movement (r=0.199; *P*<.001) Sleep duration: Week-to-week: Movement (r=0.344; *P*<.001) Daily mood (r=0.073; *P*<.001) Month-to-month Movement (r=0.676; *P*<.001) Movement: Week-to-week: Sleep (r=0.264; *P*<.001) Daily mood (r=0.295; *P*<.001) Month-to-month Sleep (r=0.663; *P*<.001) Daily mood (r=0.211; *P*<.001)		The smartphone app has the potential to provide an informative and reliable means for real-time tracking of BD status.	
**Major depressive disorder**																			
	Abbas et al [23], 2021	Canada		2021		Major depressive disorder		Digital Markers of Major Depressive Disorder (facial and vocal characteristics)		Passive (smartphone): internal camera, microphone Active: Montgomery-Asberg Depression Rating Scale (MADRS)		18		Period of collection: 4 weeks Frequency of collection: 3 time points (baseline, 2 weeks, 4 weeks)		Repeated measures ANOVA for markers from baseline to 4 weeks of antidepressant treatment. Neutral stimuli: Voice percentage (F2,26=5.6; *P*<.009) Overall expressivity (F2,28=32.6; *P*<.001) Head movement mean (F=8.9; *P*<.007) Head pose change mean (F=5.01; *P*<.033) Positive stimuli: Voice percentage (F2,26=3.59; *P*<.004) Overall expressivity (F2,28=40.67; *P*<.001) Head movement mean (F=3.58; *P*<.041) Negative stimuli: Voice percentage (F2,26=4.66; *P*<.019) Overall expressivity (F2,28=36.95; *P*<.001)		Digital markers of motor functioning associated with Major Depressive Disorder demonstrate validity as measures of response to antidepressant treatment	
	Bai et al [24], 2021	Beijing, China		2021		Major depressive disorder		Mood status and emotional stability		Passive (wearable) through smartphone app: sleep, heart rate, steps count Passive (smartphone): GPS, messages, calls, screen lock and unlock, app use Active: VAS^f^ PHQ-9^g^, GAD-7^h^		334		Period of collection: 12 weeks Frequency of collection: passive (continuously) and active (VAS: daily, PHQ-9: biweekly, GAD-7: 5 times)		The highest accuracy of classification between steady and mood swing=76.67% (SD 8.47%)		The results showed that the best model used was one in which measurements of sleep, heart rate, step count, and call logs were considered.	
	Jacobson et al [25], 2020	Lebanon, New Hampshire (United States)		2020		Major depressive disorder		Position, movement, light exposure, heart rate, cardiac variability		Passive (smartphone): GPS, Wi-Fi, Google Places, finger pressure on rear camera Active: DASS-14 (depression scale), PANAS-X^i^		31		Period of collection: 7 days Frequency of collection: passive (continuously) and active (hourly)		Correlation between predicted depressed mood levels and observed depressed mood *r*=0.587, 95% CI (0.552-0.621)		Passively collected smartphone data can accurately predict future depressed mood in a sample reporting clinical level of depression.	
	Laiou et al [26], 2022	London, United Kingdom,		2022		Major depressive disorder		Movement, socially relevant activities, environmental factors (noise and light)		Passive (smartphone): GPS, incoming and outgoing calls Active: PHQ-8		164		Period of collection: 2 years Frequency of collection: passive (continuously) and active (every 14 days)		Association between stay home and symptoms severity: Weekdays (95% CI 0.023-0.178; median 0.098; home stay: 25th-75th percentiles 17.8-22.8; median 20.9 h a day) Weekends (95% CI −0.079 to 0.149, median 0.052; home stay: 25th-75th percentiles 19.7-23.5; median 22.3 h a day)		The results suggest that staying at home is associated with the severity of major depressive disorder symptoms and illustrate that passive detection of individuals with depression is possible and may provide important clues for monitoring the course of major depressive disorder symptoms.	
	Pedrelli et al [27], 2020	United States		2020		Major depressive disorder		Smartphone use, activity levels, skin conductance, heart rate variability, sleep, social interaction		Passive (2 wristbands) through smartphone app: electrodermal activity, peripheral skin temperature, heart rate, motion from IMU^j^, and sleep from actigraphy Active: Hamilton Depression Rating Scale 17 items (HDRS-17)		31		Period of collection: 8 weeks Frequency of collection: passive (continuously,22 h a day/7 day a week) and active (6 times)		Correlation between estimate model and clinical-rated assessment = (0.46; 95% CI 0.42-0.74 to 0.7; 95% CI 0.66 to 0.74)		Monitoring patients with major depressive disorder via smartphones and wrist sensors is feasible and can provide an estimate of changes in the severity of depressive symptoms.	
	Cho et al [28], 2020	Seoul, Republic of Korea		2020		Major depressive disorder		Circadian rhythm (heart rate, activity, sleep, light exposure), mood		Passive (wearable) through smartphone application: activity, sleep, and heart rate Active: daily self-report via eMoodChart		With app CMR^k^=14 Without app CMR=59		Period of collection: 12 months Frequency of collection: passive (continuously) and active (daily)		CRM group vs non-CRM group: Total depressive episodes (n/year; exp β=.033; *P*=.03), 96.7% fewer Shorter depressive episodes (total; exp β=.005; *P*<.001), 99.5% fewer Shorter manic or hypomanic episodes (exp β=.039; *P*<.001), 96.1% Total mood episodes (exp β=.026; *P*=.008), 97.4% Shorter mood episodes (total; exp β=.011; *P*<.001), 98.9%		The results of the study confirmed that providing daily circadian rhythm–based mood prediction feedback through the CRM app with a wearable activity tracker, analyzing the life patterns of individual patients with mood disorders, significantly reduced the number and duration of mood episodes compared with those in the control group.	
**Mood disorder**																			
	Chikersal et al [29], 2021	United States		2021		Mood disorder		Depressed mood, movement, communication, smartphone use, sleep, physical activity		Passive (smartphone): Bluetooth, calls, GPS, screen Passive (wearable): steps and sleep Active: BDI-II^l^		138		Period of collection: 16 weeks Frequency of collection: passive (continuously) and active (twice, at the beginning and at the end)		Detection accuracy: Post semester depressive symptoms=85.7% Symptom severity=85.4%		Results showed that the best feature model for predicting depression was the one using a 7-feature set, including Bluetooth, calls, campus map, location, phone use, steps, and sleep.	
	Cho et al [30], 2019	Seoul, Republic of Korea		2019		Mood disorder		Mood, activity, sleep, light exposure, heart rate		Passive (smartphone): light exposure Passive (wearable) through smartphone app: sleep, activity, and heart rate Active: eMoodchart app		55		Period of collection: 2 years Frequency of collection: passive (continuously) and active (daily)		Mood state prediction accuracy for the next 3 days: All patients: 65%, AUC^m^=0.7 Major depressive disorder: 65%, AUC=0.69 Bipolar I: 64%, AUC=0.67 Bipolar II: 65%, AUC=0.67 Accuracy for all patients predictions: No episode: 85.3%, AUC=0.87 Depressive episode: 87%, AUC=0.87 Manic episode: 94%, AUC=0.958 Hypomanic episode: 91.2%, AUC=0.912		The study authors obtained a machine learning model capable of estimating the degree of accuracy of each patient’s mood status over a 3-day period, demonstrating that it is possible to develop effective learning models for predicting mood in patients with mood disorders.	
	Mehrotra et al [31], 2016	Heidelberg, Germany		2016		Mood disorder		Notification management, smartphone use, depressed mood		Passive (smartphone): number of notifications clicked, average time to view notifications, notification response time, number of apps started, time spent on apps, phone unlock count Active: PHQ-8		25		Period of collection: 30 days Frequency of collection: passive (continuously) and active (every day for 14 days)		Correlation between depression and all notification metrics of the last 14 days ranged from 0.4 to 0.6		The results suggest that using data from the last 14 days of monitoring can improve the accuracy of predicting the depressive score a user will have on the current day.	
	Wahle et al [32], 2016	Switzerland		2016		Mood disorder		Position, movement, mood		Passive (smartphone): phone use, accelerometry, Wi-Fi, GPS Active: PHQ-9		126		Period of collection: 9 months Frequency of collection: passive (continuously) and active (biweekly)		Binary classification performance for biweekly PHQ-9: Random Forest model=60.1% Support vector machine=59.1%		Results showed that participants with clinical levels of depression and who had adherence of 8 weeks or more (N=12), had lower PHQ-9 scores at the end of the study (*P*=.01). Participants who used the app for an extended period showed significant reduction in self-reported symptom severity.	

^a^RMSE: root mean square error.

^b^BD: Bipolar Disorder

^c^HC: Healthy Controls

^d^ASRM: Altman Self-Rating Mania.

^e^DASS: Depression, Anxiety and Stress Scale.

^f^VAS: Visual Analog Scale.

^g^PHQ: Patient Health Questionnaire.

^h^GAD-7: Generalized Anxiety Disorder-7.

^i^PANAS-X: Positive and Negative Affect Schedule-Expanded version.

^j^IMU: Inertial Measurement Unit

^k^CMR: Circadian Rhythm of Mood

^l^BDI-II: Beck Depression Inventory-II.

^m^AUC: Area Under the Curve.

**Table 5:** Anxiety disorder: retrieved studies and their main outcomes.

Study	Country (region)	Data	Psychiatric disorder	Symptoms investigated	Assessment technology	Sample, n	Data collection time	Statistics	Synthesis of main results
Jacobson et al [33], 2020	Lebanon, New Hampshire (United States)	2020	Social anxiety disorder	Severity of anxiety and depressive symptoms and positive and negative affects; movement and social contact	Passive (smartphone): accelerometer, incoming and outgoing calls, SMS text messages Active: SIAS (Social Interaction Anxiety Scale), DASS-21 (Depression, Anxiety, Stress Scale), self-report PANAS^a^	59	Period of collection: 2 weeks Frequency of collection: passive (continuously) and active (twice)	Correlation between predicted and observed symptoms severity: *r*=0.702	The results suggest that these passive detection data can be used to accurately predict the severity of participants’ social anxiety symptoms, specifically demonstrating a strong correlation between the predicted and observed severity of social anxiety symptoms.
Jacobson et al [34], 2021	United States	2021	Generalized Anxiety Disorder and Panic Disorder	Wake-sleep rhythms (sleep duration, wake duration, number of wake periods, and number of sleep periods), latency at sleep onset, sleep repetition time before waking up, sleep quality, and time to get up after waking up	Passive (wearable) through smartphone app: actigraphy	265	Period of collection: phase 1, phase 2: 9-14-years later phase 1; phase 3: 17-18-years later phase 1 Frequency of collection: passive (continuously for 1 week in phase 2) and active (twice in phase 1 and in phase 3)	Prediction of symptoms deterioration: (AUC^b^=0.696; 95% CI 0.598-0.793; 84.6% sensitivity; 52.7% specificity; balanced accuracy=68.7%)	The results show that through the use of wearable motion-sensing tools, such as the ActiGraph, it is indeed possible to significantly predict which individuals will experience symptom deterioration over a 17-18 years period.
Jacobson et al [35], 2022	Lebanon, New Hampshire (United States)	2022	Generalized Anxiety Disorder or Social Anxiety Disorder	Physiological activation (heart rate and heart rate variability), light exposure, social contact, and location	Passive (smartphone): GPS, Google Places, National Weather Service, finger pressure on the rear camera. Active: Self-Report of PANAS-X (positive and negative affect tab, fear, and sadness subscales) and Self-Report of MEAQ (Multidimensional Experiential Avoidance Questionnaire).	32	Period of collection: 1 week Frequency of collection: passive (continuously for 1 week) and active (hourly for 1 week)	Future changes in anxiety symptoms model: *R*^2^=0.748 Changes hour-by-hour within-person: *R*^2^=0.385	Customized deep learning models using smartphone sensor data can accurately predict future changes in anxiety disorder symptoms and even changes in the same participant from hour to hour.
Meyerhoff et al [36], 2021	Chicago, United States	2021	Mood disorder, social anxiety disorder, and Generalized Anxiety Disorder	Movement, social interactions, location	Passive (smartphone): GPS, app use, calls, messages Active: PHQ-8^c^, GAD-7 (Generalized Anxiety Disorder 7-item scale), SPIN (Social Phobia Inventory)	282	Period of collection: 16 weeks Frequency of collection: passive (every 5 min in 1 day every 3 weeks) and active (every 3 weeks)	Multimorbidity groups: changes in depression are predicted by changes in GPS features Time: *r*=−0.23; *P*=.02, locations: *r*=−0.36; *P*<.001, exercise duration: *r*=0.39; *P*=.03, and use of active apps (*r*=−0.31; *P*<.001) Depression and anxiety groups: changes in depression are predicted by changes in GPS features for locations (*r*=−0.20; *P*=.03) and transitions (*r*=−0.21; *P*=.03)	Changes in sensor-derived behavioral characteristics are associated with subsequent changes in depression, but not vice versa, suggesting a unidirectional relationship in which changes in detected behaviors are associated with subsequent changes in symptoms.

^a^PANAS: Positive and Negative Affect Schedule.

^b^AUC: area under the curve.

^c^PHQ-8: Patient Health Questionnaire-8.

**Table 6:** Substance use disorder: Retrieved studies and their main outcomes.

Study	Country (region)	Data	Psychiatric disorder	Symptoms investigated	Assessment technology	Sample, n	Data collection time	Statistics	Synthesis of main results
Epstein et al [37], 2020	Baltimore (United States)	2020	Substance use disorder	Environmental exposure	Passive (smartphone): GPS Active: questions on level of craving from heroin, cocaine, and stress level	189	Period of collection: 16 weeks Frequency of collection: passive (every 15 or 20 min) and active (at least 23 times weekly)	Overall accuracy in predicting absence of drug or stress craving=0.93% Overall accuracy in predicting presence of drug or stress craving=0.7%	The machine learning models used correctly predicted the occurrence of drug or stress craving 90 min in advance with a very good overall accuracy at the end of 16 weeks.

### Synthesized Findings

In this subsection, for each study, we report the main findings of pertinence for this systematic review. In each study, the type of technology used was specified; the main distinction was between “active” and “passive” sensing, both types of remote sensing that use digital instruments and make remote observations and measurements available, without the need for direct clinical assessment. Of the 29 studies, 2 (7%) used only instruments of active sensing, 3 (10%) used only instruments of passive sensing, and 24 (83%) used both types of instruments. In the following sections, studies were grouped according to the mental disorder of the involved patients.

#### Schizophrenia

Among the 29 studies included in the systematic review, 9 (31%) were related to schizophrenic disorders (Table 3) [10,11-18].

Abbas et al [10,11] carried out 2 studies. In the first study [10], based on the assumption that motor abnormalities were a distinct component of schizophrenia symptomatology, they developed a computer vision–based assessment of motor functioning using video data collected remotely through smartphones. They recruited 18 patients with schizophrenia and 9 healthy controls who were assessed daily for 14 days through videos recorded by the front-facing camera of the smartphone. Their computer vision model used the Euclidean distance of head movement between frames as an evaluation parameter (passive sensing); the Positive and Negative Syndrome Scale (PANSS) [38] was administered to both groups as active sensing to assess the symptoms of schizophrenia. The results showed that the rate of head movement in participants with schizophrenia (1.48 mm/frame) and in healthy controls (2.50 mm/frame) differed significantly, and a logistic regression analysis demonstrated that head movement was a significant predictor of schizophrenia diagnosis (*P*=.02), demonstrating the feasibility of using this digital marker to distinguish between participants diagnosed with schizophrenia (via gold-standard assessment: PANSS) and healthy participants. In addition, a linear regression between head movements and PANSS scores showed that head movement has a negative relationship with the severity of schizophrenia symptoms. This computer vision model can classify a schizophrenia diagnosis and quantify symptom severity and possible relapse. In the second study [11], the authors passively measured the facial and vocal characteristics of 20 patients with schizophrenia for 2 weeks through 2 classes of prompts: evoked and spontaneous; these data were related to schizophrenia symptom severity assessed using PANSS [38]. The results showed that the vocal markers were specific markers of negative symptom severity and facial expressivity was a robust marker of overall schizophrenia severity.

Adler et al [12] aimed to develop an unobtrusive remote monitoring system to detect early warning signs of the impending symptomatic relapse. They used only passive sensing data extracted from smartphones of 60 patients; the models were trained to recreate the participant behavior on days of relative health, and then a threshold was applied to predict anomalies. The collected data showed a median 108% increase in behavioral anomalies near relapse. In conclusion, this method predicted a higher rate of anomalies in patients with schizophrenia within the 30-day near-relapse period, can be used to discover changes before relapse, and can predict incipient relapse in schizophrenia.

Similar to the previous study [12], Barnett et al [13] collected passive data from smartphones to identify possible warning signs of relapse. This study was conducted on 17 patients with schizophrenia in active treatment using the Beiwe app on their smartphones for 3 months. The collected passive data were classified into 2 categories: mobility and sociability features. To identify anomalies, they first defined expected behavior through the definition of an overall trend of behavior and then tested for aberrant behavior with a statistical model on deviations from the trend. The results showed that the rate of behavioral anomalies detected 2 weeks before relapse was 71% higher than the rate of anomalies during other time periods. On this basis, the authors concluded that real-time detection of behavioral anomalies could be a determinant to act before escalation or relapse occurs.

Two studies by Henson et al [14,15] explored 2 different areas of the application of digital phenotyping to schizophrenia disorder. The first study [14] explored the relationship between active and passive sensing in individuals with schizophrenia and healthy controls. It found that 2 features of social rhythm data (ie, “Circadian Routine” and “Weekend Day Routine”), passively collected via a smartphone, were negatively associated with symptoms of anxiety, depression, psychosis, and poor sleep, actively collected via a smartphone, in patients; in healthy controls, more stable social rhythms were positively related to symptoms. These differences showed that passive sensing can be used to understand how daily routine affects symptoms in patients with schizophrenia and allowed us to distinguish between participants clinically diagnosed with schizophrenia (via clinical gold-standard assessment) and healthy participants. The second study [15] sought to predict the occurrence of relapse in patients with schizophrenia by detecting anomalies in the active and passive data. The authors provided a model composed of 6 features (both active and passive sensing) that were studied longitudinally for each patient and for healthy controls to establish a baseline and then tested simultaneously for anomalies (ie, significant deviation from baseline). The overall investigation resulted in 89% sensitivity and 75% specificity for predicting relapse in patients with schizophrenia.

Ranjan et al [16] conducted a longitudinal study to evaluate the association between symptom changes and home time in patients with schizophrenia. They enrolled 86 patients and recorded both passive and active data through a smartphone app (mindLAMP) for 6 months. The results showed a high correlation between self-reported symptoms and gold-standard clinical assessment scores. In conclusion, the data demonstrated the utility of this method as a diagnostic tool and potentially as a tool for tailoring treatment. Strauss et al [17], to overcome some of the limitations of negative symptom assessment in schizophrenia, evaluated the validity of accelerometry as a passive method of collecting data on the presence, vigor, and variability of movement. They enrolled 50 patients with schizophrenia who were demographically matched to 70 healthy controls. Each participant underwent surveys as an active assessment (eg, PANSS; Structured Clinical Interview for Diagnostic and Statistical Manual of Mental Disorders, Fifth Edition; and Brief Negative Symptom Scale) and passively recorded data regarding accelerometry via smartphones. Patients with schizophrenia had lower scores on vigor and variability of movement than healthy controls. The variables extracted from accelerometry demonstrated convergent validity with active data obtained from negative symptoms surveys (gold-standard assessment: PANSS), demonstrating that this approach could support gold-standard assessment. In conclusion, accelerometry is a valid objective measure of negative symptoms.

The latest study on schizophrenia was conducted by Wang et al [18] based on the premise that early detection of changes in the mental status of individuals with severe mental illness is critical for effective intervention. This study involved 21 patients with a clinical diagnosis of schizophrenia who were recently discharged from the hospital who were passively monitored with a smartphone (CrossCheck sensing system) that collected data on sleep, mobility, conversations, and smartphone use. At the same time, every patient had to answer 10 questions about positive and negative symptoms every 2 or 3 days. The results indicated a statistically significant association between the automatically tracked behavioral features related to sleep, mobility, conversations, smartphone use (passive data), and self-reported indicators of mental health (active data).

#### Mood Disorders

Of the 29 studies, 15 (52%) involved ≥1 mood disorders (Table 4) [19-24,25-32,39]. In the following subsections, studies concerning mood disorders have been divided into 3 sections based on the mood disorder considered.

##### Bipolar Disorder

Of these 15 studies, 4 (27%) studies were specifically related to bipolar disorder.

Busk et al [19] provided an Android smartphone app to 84 patients with bipolar disorder who had to answer a self-report questionnaire for approximately 3 years. In addition, patients with bipolar disorder were assessed by trained psychiatrists using the Hamilton Depression Rating Scale (HDRS; Hamilton [40]) and the Young Mania Rating Scale (Young et al [41]). This study aimed to examine the feasibility of predicting daily subjective mood scores based on patients’ daily self-assessments. Applying a Bayesian hierarchical regression model, it was observed that a 4-day self-assessment history can predict future mood scores.

In the second study, Ebner-Priemer et al [20] monitored 29 patients with bipolar disorder over a 12-month period using the mobile sensing module of movisensXS to track data from different smartphone sensors along with a biweekly evaluation provided by experienced clinicians. Using a structural equation model, we identified 2 latent psychopathological outcomes (mania and depression) and 3 latent phenotype predictors (sleep, activity, and communicativeness). The results showed that a reduction in sleep duration and an increase in activity were associated with a higher level of mania (β=−.098 and β=.123, respectively); on the other hand, the only efficient predictor of depression was activity: days with more activity than average correlated with lower levels of depression. This study has shown that it is possible to identify digital phenotype patterns in the days preceding a psychopathological episode, which can be used as digital prodromal predictors.

Tseng et al [22] tracked the mood, sleep, and activity levels of 159 patients with bipolar disorder to monitor their status. They collected both active and passive data from a smartphone app: active data were obtained from Young Mania Rating Scale [41], HDRS [40], Altman Self-Rating Mania [42], and Depression, Anxiety and Stress Scale-21 items (DASS-21 [43]), and passive data were recorded via GPS sensors. The results showed that mood, sleep, and activity levels correlated with the same data on the next day, the next week, and the next month. These findings support the idea that this smartphone app provides an informative and reliable tool for the real-time monitoring of bipolar status.

The latest study on bipolar disorder by Faurholt-Jepsen et al [21] combined active and passive sensing technologies to investigate mobility differences between patients with bipolar disorder and healthy participants and to investigate mobility differences in participants with bipolar disorder in different affective phases. The results showed that participants with bipolar disorder had lower location entropy compared with healthy participants (β=−.14; *P*=.009), were less mobile during a depressive state compared with a euthymic state, and had lower location entropy during an affective state (depression or mania) compared with a euthymic state. The authors concluded that alterations in location data reflecting mobility patterns may be a promising measure of illness and illness activity in participants with bipolar disorder.

##### Major Depressive Disorder

Of the 15 studies, 6 (40%) were related only to major depressive disorder. Jacobson and Chung [25] attempted to predict depressive mood hour-by-hour in a sample of 31 college students with major depressive disorder. Each participant installed a mobile phone app called “Mood Triggers” that recorded location, social, and heart rate information (passive data); at the same time, every hour, participants had to fill out 2 self-assessment questionnaires (active data: DASS-21 [43] and Positive and Negative Affect Schedule-Expanded version [PANAS-X] [44]). The results showed a moderate and significant correlation between active and passive data (*r*=0.587) and demonstrated that it was possible to predict, through passive data collected by a smartphone, the future depressed state of a sample reporting clinical levels of depression, demonstrating that digital phenotyping approach could support gold-standard assessment (DASS-21 and PANAS-S) in symptoms evaluation.

Abbas et al [23], based on the assumption that changes in facial expressions and verbal production in patients with depression could correspond to the level of severity of the disorder, conducted a study to test whether these digitally detected markers could be used as measures of treatment validity. They enrolled 18 patients with major depressive disorder who were treated for 4 weeks with selective serotonin reuptake inhibitors or serotonin or norepinephrine reuptake inhibitors; during these 4 weeks, patients underwent biweekly checks by administration of a psychometric scale (Montgomery-Asberg Depression Rating Scale [45]) and passive measurements using the AiCure app. Through video responses lasting at least 10 seconds, several markers related to the measurement of facial, vocal, and movement behaviors and their changes over time were observed. The results demonstrated a consistent effect of antidepressant treatment on digital markers that were highly concordant with the symptomatic changes.

Bai et al [24] examined the feasibility of passive monitoring of changes in depression levels in patients with major depressive disorder. They enrolled 334 participants and recorded information about heart rate, sleep, number of steps, call logs, SMS text message logs, app use, GPS, and screen on/off status through an Android app connected with an Mi Band 2 (Xiaomi Corporation); at the same time, participants answered daily self-assessment questionnaires (Patient Health Questionnaire-9 [PHQ-9] [46], Visual Analog Scale [47], and Generalized Anxiety Disorder-7 [GAD-7] [48]). Using these data, different clusters of depressed mood (from stable to fluctuating) were identified and different features were extracted. These features, combined through 6 machine learning models, enabled the identification of the best behaviors to classify the different behavioral states of the patients, demonstrating the feasibility of using digital data acquired through smartphones for supporting gold-standard assessments (PHQ-9, Visual Analog Scale, and GAD-7). The results indicated that the best model with a total accuracy of 75% was the one in which sleep, heart rate, number of steps, and call logs were considered together.

Pedrelli et al [27] conducted a study to evaluate the feasibility of using passive and active sensing technologies to detect levels of depressive symptom severity. Overall, 31 participants with major depressive disorder were passively monitored for 8 weeks with smartphone sensors and 2 wearable wristbands, obtaining information on electrodermal activity, peripheral skin temperature, heart rate, movement, and sleep. Information on social interaction, activity, and app use was recorded through a smartphone app (movisensXS). During these 8 weeks, each participant underwent 6 clinical interviews, including the administration of the Hamilton Depression Rating Scale-17 (HDRS-17 [40]). Using passive data, 3 machine learning models were developed to predict symptoms, 1 combined (smartphone and wristbands) and 2 singulars (only smartphone or only wristbands). The results indicated a correlation between active data and these 3 models (passive data) that ranged from moderate to high (*r* ranged from 0.46 to 0.70), showing that passive data models could support gold-standard assessment (HDRS).

Cho et al [28] carried out a prospective study in a sample of 73 participants affected by major depressive disorder to evaluate the feasibility of a smartphone app called “Circadian Rhythm for Mood.” The app sent alerts when abnormal behavior was detected based on a deviation from the usual life pattern, and participants had to fill out a mood self-evaluation scale (eMoodChart) daily. The results of the analysis of individual life patterns for each participant showed that sending daily mood feedback, estimated based on circadian rhythm, led to a reduction in symptoms by 97.4% and a decrease in their duration by 98.9%.

The last study investigating major depressive disorder was conducted by Laiou et al [26] to relate reported depressive symptoms and time spent at home in a group of 164 patients. Each patient had to complete the PHQ-9 [46] biweekly to collect data on their mood (active sensing), while information on GPS location, social activity, and movement was passively collected continuously. Using linear regression, the authors could associate the time spent at home and symptoms severity with sex, age, and occupational state as confounding variables. The results showed that more time spent at home correlated with older age and greater symptom severity and the relationship between more time spent at home and greater symptom severity was stronger on weekdays than on holidays.

##### Mood Disorder

The last 5 of 14 studies on mood disorders group 2 sample classes. They studied participants with major depressive disorder or bipolar disorder [30] or participants without any clinical diagnosis of major depressive disorder that however emerged as clinically depressed during the study [29,31,32,39].

Only 1 study, carried out by Cho et al [30], enrolled both patients with bipolar disorder and patients with major depressive disorder. The aim of this study was to evaluate whether through the observation of circadian rhythm it was possible to predict each patient’s mood. The authors used a mobile app combined with a wearable wristband (Fitbit Charge, Fitbit Inc) to passively collect data on sleep, activity, light exposure, and heart rate for 2 years. Simultaneously, every patient had to fill out a self-assessment questionnaire on their health status (active data). The authors obtained a model that estimated mood with 65% accuracy for both bipolar disorder and major depressive disorder.

Canzian and Musolesi [39] attempted to predict a depressive state in people by analyzing the mobility behavior of 28 people with depressive disorder. They provided participants with a mobile smartphone app called “MoodTraces” that recorded location information through GPS sensors and provided daily mood questionnaires. This information was put together by inferential algorithms demonstrating that based on mobility pattern and depressed mood, they could predict the status of patients with a mood disorder. Chikersal et al [29] conducted a study on college students to monitor their depressive state during one semester. Each student downloaded a smartphone app to passively collect data on location and phone use and was provided with a FitBit Flex 2 to monitor sleep and step counting. Finally, all participants completed the Beck Depression Inventory-II [49] at the beginning and end of the semester. After testing several models, the best model was based on a set of 7 features: Bluetooth (calculated from the scanned Bluetooth addresses recorded by the Bluetooth sensor), phone call (calculated using the call logs), campus map (analysis of location patterns in relation to the college campus), location (derived from a proprietary algorithm that estimates location based on GPS, Wi-Fi, and cell tower signals), phone use (calculated using the screen status sensor, which recorded screen status: on, off, lock, and unlock), steps (calculated from the step count over time returned by the Fitbit application programming interface), and sleep (calculated from the sleep inferences over time returned by the Fitbit application programming interface). This model could predict depressive symptoms with >80% accuracy and 11 to 15 weeks in advance.

Mehrotra et al [31] conducted a longitudinal study designed to predict depressive state using various data collected passively through smartphone sensors, after which participants were required to fill out the PHQ-8 [46] daily for 30 days in a row. The results showed that by using data from the previous 14-day data, it was possible to predict the depressive score on the current day.

The last study on mood disorders was conducted by Wahle et al [32] to identify participants with a clinical level of depression and to evaluate the potential for context-sensitive interventions. Each participant downloaded a smartphone app called “MOSS” that passively collected information on physical activity, social, and location, and they also had to fill out a self-evaluation questionnaire (PHQ-9 [46]) biweekly. The app has been able to provide participants with targeted cognitive behavioral interventions, predicting symptomatic worsening and thus avoiding the relapse.

#### Anxiety Disorders

Of the 25 studies, 4 (16%) concerned anxiety disorders (Table 5) [33,34-36]. Jacobson et al [33,34,35] conducted 3 studies. The first study [33] aimed to predict the severity of social anxiety in a group of 59 participants through a smartphone app (“Sensus”) that passively collected data on mobility and social behavior, and in addition, each participant had to fill 3 self-evaluation questionnaires (DASS-21 [43]; Social Interaction Anxiety Scale—Mattick and Clarke [50]; PANAS [44]). The results showed a strong correlation (*r*=0.702; *P*<.001) between the severity of expected social anxiety symptoms (extracted from data collected through the smartphone app) and the severity of observed social anxiety symptoms, showing convergent validity between the gold-standard assessment (PANAS) and data acquired through smartphones. The second study [34] sought to predict long-term exacerbation of symptoms in a sample of 265 participants with GAD or panic disorder. In this study, passive data on movement were collected using an ActiGraph, and active data were collected through a structured interview. The results indicated that long-term symptom exacerbation could be significantly predicted with a sensitivity of 84.6% and a specificity of 52.5% in a period of 16 to 17 years (study duration). The third study [35] evaluated a sample of 32 participants with GAD to observe whether passive data collected through the smartphone could accurately predict momentary changes in anxiety and avoidance symptoms. Each participant filled 2 self-questionnaires (multidimensional experiential avoidance questionnaire [51] and PANAS-X [44]) through a smartphone app that simultaneously collected information on physiological activation, sociality, light exposure, and location (GPS). Results showed that by using these data, it was possible to predict most changes in anxiety symptoms (𝑅^2^=0.748) and also changes in symptoms in the same participant hour-by-hour (media 𝑅^2^=0.385). The last study concerning anxiety disorders was carried out by Meyerhoff et al [36]. The authors conducted a longitudinal cohort study in which they collected passive data from smartphones on location (GPS), communication, and app use. The sample comprised 282 participants with depression or social anxiety or generalized anxiety, and the aim was to predict changes in depressive symptoms in participants with anxiety disorders. The results showed that behavioral changes detected passively through smartphones were associated with symptomatic changes.

#### Substance Use Disorder

The only study concerning substance use disorder was conducted by Epstein et al [37], who sought to predict drug craving or stress level in a sample of 189 patients with substance use disorder. Movements of each participant were monitored through a wearable wristband (BT-Q1000X), and each participant installed an app on their smartphone that sent an alert 3 times a day in response to which the participant had to indicate their level of craving and stress. After 16 weeks of observation, machine learning models accurately predicted drug craving or stress 90 minutes in advance: the absence of drug craving or stress was predicted with 0.93% accuracy, whereas the presence of drug craving or stress was predicted with 0.70% accuracy.

## Discussion

### Overview

This systematic review aimed to evaluate the potential of digital phenotyping as an innovative new approach to monitor patients with mental health disorders and to determine whether it could predict relapse or symptomatologic exacerbations. The included studies had 2 possible general objectives: either they coped with symptom estimation through validated passive data to bypass the already sparse medical examinations (this was the most common aim) or they aimed at the prediction of symptoms over time, and the sign detected with the digital phenotype approach today should predict a symptom or a relapse in the future. The first purpose, if achieved, would allow for continuous health monitoring and thus immediate intervention; the second purpose might even lead to the implementation of preventive approaches.

In analyzing the studies included in our systematic review, it is possible to observe that they range widely among disorders, but they also strongly focus on mood disorders, which is in line with the high prevalence rate of these disorders in the general population. The other most studied disorder was schizophrenia (9 studies), followed by anxiety (4 studies), and substance use disorder (only 1 study). All the studies have been carried out in developed countries with a high degree of technological development, such as the United States, China, the United Kingdom, Taiwan, and Germany, and none of the studies were conducted in low-income countries, demonstrating how the progress of medicine is not equally distributed across the world.

The COVID-19 pandemic, especially the lockdown, exacerbated the burden of these disorders [52]. In addition, the disruption of many health services, associated with the pandemic restrictions, has worsened the situation, making it increasingly necessary to move toward telemedicine. Accordingly, this research line is growing rapidly, as shown by the publication year of the included studies: 79% (23/29) of the studies have been published from 2020 to 2022, proving that the flaws caused by the pandemic in the health care system have accelerated experimentation with digital phenotype–based approaches in medicine.

### Principal Outcomes

#### Digital Phenotyping and Schizophrenia

The most dangerous risk in schizophrenia is relapse, which can occur even months after discharge from the hospital; therefore, continuous monitoring and prediction of symptom exacerbation are key tools to early treatment of patients, with the goal of reducing hospitalization and consequently the cost of care. Through physiological and behavioral passive data collected via smartphone apps, such as CrossCheck [12,18], mindLAMP [16], Beiwe [13], or wearable wristbands (Embrace Band) [17], it has been possible to predict the onset of relapse very effectively. Furthermore, relapses in schizophrenia are characterized by clear physiological and social signals; therefore, Abbas et al [10,11], by observing head movements in patients with schizophrenia [10] and by measuring facial and vocal characteristics [11], have very successfully quantified the severity of negative symptoms and predicted exacerbation. Finally, the study of changes in behavioral outcomes has shown that some variables related to the daily routine can be good predictors of relapse [14], and the longitudinal study of behavioral variables (mobility, sociality, sleep, etc) generated models that can accurately predict relapses [15]. Hence, digital phenotyping allows the prediction of relapse and consequently may offer the potential for immediate interventions to avoid hospitalizations.

#### Digital Phenotyping and Mood Disorders

There were 15 studies concerning mood disorders: major depressive disorder (6/15, 40%), characterized by depressed mood, poor mobility, insomnia or hypersomnia, and social retirement; bipolar disorder (4/15, 27%), characterized by alternating depressed mood and manic mood; and mood disorders in general (5/15, 33%). These studies showed that all of these behavioral outcomes can be easily detected using digital phenotyping as all features related to movement and social rhythm can be recorded through smartphone sensors (eg, GPS) or wearable wristbands, and it was found that passive data were correlated with active data [25-27,32]. Besides, passive data can be used to predict future mood and, therefore, any relapse or worsening of symptoms in patients with mood disorders; using wearable wristbands, Bai et al [24] and Pedrelli et al [27] collected information on sleep, step count, and heart rate, and through machine learning algorithms developed models capable of predicting changes in depressed mood; Jacobson and Chung [25] and Laiou et al [26], using smartphones, focused on mobility and sociality to predict future depressed mood [25] and symptoms worsening [26]; and the combination of smartphones and wearable wristbands, used by Cho et al [30] to collect data on sleep, activity, light exposure, and heart rate, showed that it was possible to predict mood over 3 days. Summarizing all studies related to mood disorders, the most widely used class of features for predicting or discriminating symptoms is mobility and activity; for example, Ebner-Priemer et al [20] observed that higher levels of mobility are associated with higher levels of mania; Faurholt-Jepsen et al [21] observed reduced mobility patterns during depressive states; Laiou et al [26] noted that the more time spent at home, the more severe the symptoms; and Tseng et al [22], by combining active (mood and sleep) and passive data (activity), demonstrated that it is possible to obtain real-time monitoring and predict changes in the symptoms of a patient with bipolar disorder using a smartphone app.

#### Digital Phenotyping and Anxiety Disorders

Anxiety disorders are characterized by great variability in symptomatology, and this peculiarity is reflected in the heterogeneity of the studies included in the systematic review because each of them focuses on different disorders, such as social anxiety disorder, panic disorder, and GAD. As with mood disorders, digital phenotyping is an effective approach for estimating symptoms through passively collected data via smartphones as they are correlated with active data. Meyerhoff et al [36] showed that changes in behavior detected through smartphone sensors were correlated with changes in patients symptoms. Regarding symptom prediction, 3 different studies have shown that it is possible to effectively predict most changes in anxiety symptoms and even changes in the same participant hour-by-hour through data collected via smartphone sensors [33,35] and data collected through ActiGraph [34].

#### Digital Phenotyping and Substance Use Disorder

Only 1 study [37] has been included in our systematic review regarding substance use disorder; probably the reason is that individuals with this disorder are very difficult to “hook” and persuade to participate in such studies, but precisely because of the recursive nature of addiction, it would be very important to observe participants and predict relapses; therefore, Epstein et al [37] built a model that can effectively predict the absence of need for drugs or stress 90 minutes in advance. However, the paucity of studies does not allow us to say with certainty whether the digital phenotyping approach can be used to estimate the symptoms of this disorder or to predict possible relapse.

### Limitations

Our systematic review has some limitations that need to be addressed. The samples chosen in the included studies were limited and were mostly clinical samples; moreover, the study design often differed among studies, which did not allow the possibility of generalizing the results to the general population. The possibility of a future attempt at generalization opens up the privacy issue; indeed, although it has so far been easy to obtain informed consent from experimental participants because they were few and enrolled in a clinical setting, when trying to expand the use of digital phenotyping to larger and nonclinical populations, gaining access to sensitive data of so many participants will be a difficult challenge to overcome, both because of a concern to share so much information regarding their private life and because they may be distrustful of the good use that will be made of the shared data. Another issue to consider is the objectivity of self-report questionnaires, which are used in the convergent validation process of this new approach and could sometimes make this process less reliable because the answers to the questionnaires are given purely subjectively by participants with mental disorders, and sometimes their judgments could be “flawed” by the pathology itself.

### Conclusions

In conclusion, our systematic review confirms the feasibility of using this new instrument, digital phenotyping, to predict symptomatic changes in patients with mental disorders. Taken together, the results for each mental disorder are consistent and indicate the effectiveness of digital phenotyping in the mental health field to support the gold-standard assessment. Given the consistency of this approach, it would be very useful in the future to use it for mental disorders that have not yet been sufficiently covered, such as eating disorders, which would require almost constant monitoring of the participant to avoid risk behaviors, such as binge eating or self-induced vomiting.

**Figure 3:**
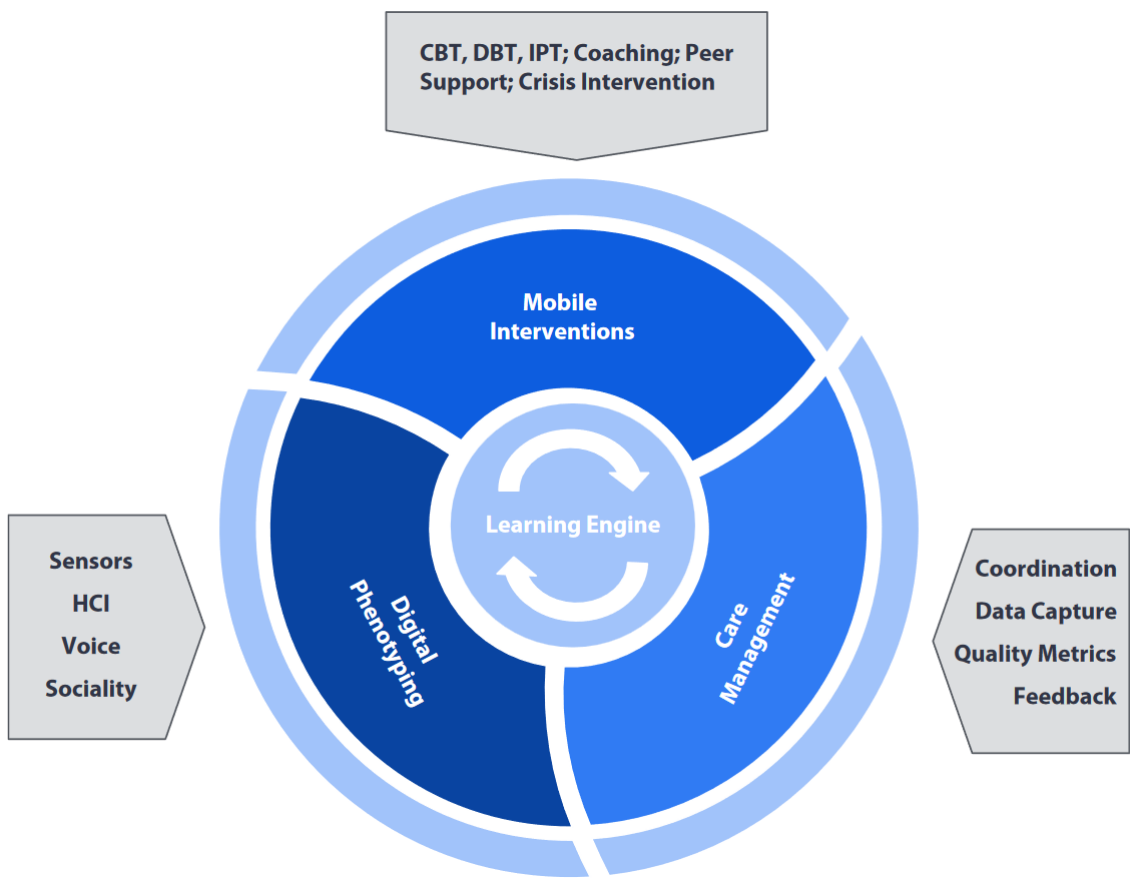
New health care model: it integrates remote interventions (mobile interventions) with the use of digital phenotyping and remote general health care management (care management) to achieve moment-by-moment control of the patient and assist them throughout the diagnosis and treatment process [53]. CBT: cognitive behavioral therapy; DBT: dialectical behavior therapy; HCI: human computer interaction; IPT: interpersonal therapy.

For future studies, researchers should consider that, for example, mobility features have been shown to be the most reliable for disorders where mobility is affected (depression, schizophrenia, etc); thus, based on this assumption, an association should always be sought between behavioral outcomes of the mental disorders and how these could be reflected in the use of technologies. These possible associations should form the basis of the study designs in this area. At this experimental stage, this approach, owing to the diffusion of smartphones with digital phenotyping equipment, has the potential to result in large-scale effects that can bridge the gap between the growing need for mental health intervention and the insufficient reach of health care. Through digital devices, a new model of health care could be achieved by integrating symptom measurement (digital phenotyping), tailored mobile interventions (cognitive behavioral therapy–based interventions or interventions to manage crises during symptom exacerbation), and care management. The combination of these 3 aspects would allow direct and constant monitoring of the patient’s health status, with the possibility of creating adaptive and tailored interventions that take into account moment-by-moment changes in symptoms, while also leading to large savings in treatment costs [53] (Figure 3).

## Appendix

Multimedia Appendix 1 PRISMA (Preferred Reporting Items for Systematic Reviews and Meta-Analyses) checklist [7,8].

Multimedia Appendix 2 PRISMA (Preferred Reporting Items for Systematic Reviews and Meta-Analyses) abstract checklist [8].

Multimedia Appendix 3 Appraisal tool for cross-sectional studies (AXIS [9]).

## Abbreviations

DASS-21: Depression, Anxiety and Stress Scale–21 items
GAD: Generalized Anxiety Disorder
HDRS: Hamilton Depression Rating Scale
PANAS: Positive and Negative Affect Schedule
PANSS: Positive and Negative Syndrome Scale
PHQ: Patient Health Questionnaire
PICOS: Population, Intervention, Comparison, Outcomes, and Study Design
PRISMA: Preferred Reporting Items for Systematic Reviews and Meta-Analyses
YMRS: Young Mania Rating Scale

## References

[1] (2022). Mental health and COVID-19: early evidence of the pandemic’s impact. World Health Organization.

[2] (2022). Third round of the global pulse survey on continuity of essential health services during the COVID-19 pandemic: November–December 2021. World Health Organization.

[3] Insel TR (2017). Digital phenotyping: technology for a new science of behavior. JAMA.

[4] Insel TR (2018). Digital phenotyping: a global tool for psychiatry. World Psychiatry.

[5] Insel TR (2018). Technology as a tool for mental disorders-reply. JAMA.

[6] Montag C, Sindermann C, Baumeister H (2020). Digital phenotyping in psychological and medical sciences: a reflection about necessary prerequisites to reduce harm and increase benefits. Curr Opin Psychol.

[7] Moher D, Liberati A, Tetzlaff J, Altman DG, PRISMA Group (2009). Preferred reporting items for systematic reviews and meta-analyses: the PRISMA statement. PLoS Med.

[8] Page MJ, McKenzie JE, Bossuyt PM, Boutron I, Hoffmann TC, Mulrow CD, Shamseer L, Tetzlaff JM, Akl EA, Brennan SE, Chou R, Glanville J, Grimshaw JM, Hróbjartsson A, Lalu MM, Li T, Loder EW, Mayo-Wilson E, McDonald S, McGuinness LA, Stewart LA, Thomas J, Tricco AC, Welch VA, Whiting P, Moher D (2021). The PRISMA 2020 statement: an updated guideline for reporting systematic reviews. BMJ.

[9] Downes MJ, Brennan ML, Williams HC, Dean RS (2016). Development of a critical appraisal tool to assess the quality of cross-sectional studies (AXIS). BMJ Open.

[10] Abbas A, Yadav V, Smith E, Ramjas E, Rutter SB, Benavidez C, Koesmahargyo V, Zhang L, Guan L, Rosenfield P, Perez-Rodriguez M, Galatzer-Levy IR (2021). Computer vision-based assessment of motor functioning in schizophrenia: use of smartphones for remote measurement of schizophrenia symptomatology. Digit Biomark.

[11] Abbas A, Hansen BJ, Koesmahargyo V, Yadav V, Rosenfield PJ, Patil O, Dockendorf MF, Moyer M, Shipley LA, Perez-Rodriguez MM, Galatzer-Levy IR (2022). Facial and vocal markers of schizophrenia measured using remote smartphone assessments: observational study. JMIR Form Res.

[12] Adler DA, Ben-Zeev D, Tseng VW, Kane JM, Brian R, Campbell AT, Hauser M, Scherer EA, Choudhury T (2020). Predicting early warning signs of psychotic relapse from passive sensing data: an approach using encoder-decoder neural networks. JMIR Mhealth Uhealth.

[13] Barnett I, Torous J, Staples P, Sandoval L, Keshavan M, Onnela JP (2018). Relapse prediction in schizophrenia through digital phenotyping: a pilot study. Neuropsychopharmacology.

[14] Henson P, Barnett I, Keshavan M, Torous J (2020). Towards clinically actionable digital phenotyping targets in schizophrenia. NPJ Schizophr.

[15] Henson P, D'Mello R, Vaidyam A, Keshavan M, Torous J (2021). Anomaly detection to predict relapse risk in schizophrenia. Transl Psychiatry.

[16] Ranjan T, Melcher J, Keshavan M, Smith M, Torous J (2022). Longitudinal symptom changes and association with home time in people with schizophrenia: an observational digital phenotyping study. Schizophr Res.

[17] Strauss GP, Raugh IM, Zhang L, Luther L, Chapman HC, Allen DN, Kirkpatrick B, Cohen AS (2022). Validation of accelerometry as a digital phenotyping measure of negative symptoms in schizophrenia. Schizophrenia (Heidelb).

[18] Wang R, Aung MS, Abdullah S, Brian R, Campbell AT, Choudhury T, Hauser M, Kane J, Merrill M, Scherer EA, Tseng VW, Ben-Zeev D (2016). CrossCheck: toward passive sensing and detection of mental health changes in people with schizophrenia. Proceedings of the 2016 ACM International Joint Conference on Pervasive and Ubiquitous Computing.

[19] Busk J, Faurholt-Jepsen M, Frost M, Bardram JE, Vedel Kessing L, Winther O (2020). Forecasting mood in bipolar disorder from smartphone self-assessments: hierarchical Bayesian approach. JMIR Mhealth Uhealth.

[20] Ebner-Priemer UW, Mühlbauer E, Neubauer AB, Hill H, Beier F, Santangelo PS, Ritter P, Kleindienst N, Bauer M, Schmiedek F, Severus E (2020). Digital phenotyping: towards replicable findings with comprehensive assessments and integrative models in bipolar disorders. Int J Bipolar Disord.

[21] Faurholt-Jepsen M, Busk J, Vinberg M, Christensen EM, Frost M, Bardram JE, Kessing LV, HelgaÞórarinsdóttir (2021). Daily mobility patterns in patients with bipolar disorder and healthy individuals. J Affect Disord.

[22] Tseng YC, Lin EC, Wu CH, Huang HL, Chen PS (2022). Associations among smartphone app-based measurements of mood, sleep and activity in bipolar disorder. Psychiatry Res.

[23] Abbas A, Sauder C, Yadav V, Koesmahargyo V, Aghjayan A, Marecki S, Evans M, Galatzer-Levy IR (2021). Remote digital measurement of facial and vocal markers of major depressive disorder severity and treatment response: a pilot study. Front Digit Health.

[24] Bai R, Xiao L, Guo Y, Zhu X, Li N, Wang Y, Chen Q, Feng L, Wang Y, Yu X, Xie H, Wang G (2021). Tracking and monitoring mood stability of patients with major depressive disorder by machine learning models using passive digital data: prospective naturalistic multicenter study. JMIR Mhealth Uhealth.

[25] Jacobson NC, Chung YJ (2020). Passive sensing of prediction of moment-to-moment depressed mood among undergraduates with clinical levels of depression sample using smartphones. Sensors (Basel).

[26] Laiou P, Kaliukhovich DA, Folarin AA, Ranjan Y, Rashid Z, Conde P, Stewart C, Sun S, Zhang Y, Matcham F, Ivan A, Lavelle G, Siddi S, Lamers F, Penninx BW, Haro JM, Annas P, Cummins N, Vairavan S, Manyakov NV, Narayan VA, Dobson RJ, Hotopf M (2022). The association between home stay and symptom severity in major depressive disorder: preliminary findings from a multicenter observational study using geolocation data from smartphones. JMIR Mhealth Uhealth.

[27] Pedrelli P, Fedor S, Ghandeharioun A, Howe E, Ionescu DF, Bhathena D, Fisher LB, Cusin C, Nyer M, Yeung A, Sangermano L, Mischoulon D, Alpert JE, Picard RW (2020). Monitoring changes in depression severity using wearable and mobile sensors. Front Psychiatry.

[28] Cho CH, Lee T, Lee JB, Seo JY, Jee HJ, Son S, An H, Kim L, Lee HJ (2020). Effectiveness of a smartphone app with a wearable activity tracker in preventing the recurrence of mood disorders: prospective case-control study. JMIR Ment Health.

[29] Chikersal P, Doryab A, Tumminia M, Villalba DK, Dutcher JM, Liu X, Cohen S, Creswell KG, Mankoff J, Creswell JD, Goel M, Dey AK (2021). Detecting depression and predicting its onset using longitudinal symptoms captured by passive sensing: a machine learning approach with robust feature selection. ACM Trans Comput Hum Interact.

[30] Cho CH, Lee T, Kim MG, In HP, Kim L, Lee HJ (2019). Mood prediction of patients with mood disorders by machine learning using passive digital phenotypes based on the circadian rhythm: prospective observational cohort study. J Med Internet Res.

[31] Mehrotra A, Hendley R, Musolesi M (2016). Towards multi-modal anticipatory monitoring of depressive states through the analysis of human-smartphone interaction. Proceedings of the 2016 ACM International Joint Conference on Pervasive and Ubiquitous Computing: Adjunct.

[32] Wahle F, Kowatsch T, Fleisch E, Rufer M, Weidt S (2016). Mobile sensing and support for people with depression: a pilot trial in the wild. JMIR Mhealth Uhealth.

[33] Jacobson NC, Summers B, Wilhelm S (2020). Digital biomarkers of social anxiety severity: digital phenotyping using passive smartphone sensors. J Med Internet Res.

[34] Jacobson NC, Lekkas D, Huang R, Thomas N (2021). Deep learning paired with wearable passive sensing data predicts deterioration in anxiety disorder symptoms across 17-18 years. J Affect Disord.

[35] Jacobson NC, Bhattacharya S (2022). Digital biomarkers of anxiety disorder symptom changes: personalized deep learning models using smartphone sensors accurately predict anxiety symptoms from ecological momentary assessments. Behav Res Ther.

[36] Meyerhoff J, Liu T, Kording KP, Ungar LH, Kaiser SM, Karr CJ, Mohr DC (2021). Evaluation of changes in depression, anxiety, and social anxiety using smartphone sensor features: longitudinal cohort study. J Med Internet Res.

[37] Epstein DH, Tyburski M, Kowalczyk WJ, Burgess-Hull AJ, Phillips KA, Curtis BL, Preston KL (2020). Prediction of stress and drug craving ninety minutes in the future with passively collected GPS data. NPJ Digit Med.

[38] Kay SR, Fiszbein A, Opler LA (1987). The positive and negative syndrome scale (PANSS) for schizophrenia. Schizophr Bull.

[39] Canzian L, Musolesi M (2015). Trajectories of depression: unobtrusive monitoring of depressive states by means of smartphone mobility traces analysis. Proceedings of the 2015 ACM International Joint Conference on Pervasive and Ubiquitous Computing.

[40] Hamilton M (1960). A rating scale for depression. J Neurol Neurosurg Psychiatry.

[41] Young RC, Biggs JT, Ziegler VE, Meyer DA (1978). A rating scale for mania: reliability, validity and sensitivity. Br J Psychiatry.

[42] Altman EG, Hedeker D, Peterson JL, Davis JM (1997). The Altman self-rating mania scale. Biol Psychiatry.

[43] Lovibond PF, Lovibond SH (1995). The structure of negative emotional states: comparison of the depression anxiety stress scales (DASS) with the beck depression and anxiety inventories. Behav Res Ther.

[44] Watson D, Clark LA, Tellegen A (1988). Development and validation of brief measures of positive and negative affect: the PANAS scales. J Pers Soc Psychol.

[45] Montgomery SA, Asberg M (1979). A new depression scale designed to be sensitive to change. Br J Psychiatry.

[46] Löwe B, Kroenke K, Herzog W, Gräfe K (2004). Measuring depression outcome with a brief self-report instrument: sensitivity to change of the patient health questionnaire (PHQ-9). J Affect Disord.

[47] Price DD, McGrath PA, Rafii A, Buckingham B (1983). The validation of visual analogue scales as ratio scale measures for chronic and experimental pain. Pain.

[48] Spitzer RL, Kroenke K, Williams JB, Löwe B (2006). A brief measure for assessing generalized anxiety disorder: the GAD-7. Arch Intern Med.

[49] Beck AT, Steer RA, Brown GK (1996). Manual for the Beck Depression Inventory-II.

[50] Mattick RP, Clarke JC (1998). Development and validation of measures of social phobia scrutiny fear and social interaction anxiety. Behav Res Ther.

[51] Gámez W, Chmielewski M, Kotov R, Ruggero C, Watson D (2011). Development of a measure of experiential avoidance: the multidimensional experiential avoidance questionnaire. Psychol Assess.

[52] COVID-19 Mental Disorders Collaborators (2021). Global prevalence and burden of depressive and anxiety disorders in 204 countries and territories in 2020 due to the COVID-19 pandemic. Lancet.

[53] Insel TR (2019). Bending the curve for mental health: technology for a public health approach. Am J Public Health.

