# Supplementary material for: Digital Phenotyping for Monitoring Mental Disorders: Systematic Review
Source: J Med Internet Res. 2023 Dec 13;25:e46778. doi: 10.2196/46778 (PMC10753422; doi:10.2196/46778)
Supplement: Multimedia Appendix 3 [file jmir_v25i1e46778_app3.docx]

| **The Appraisal tool for Cross-Sectional Studies (AXIS tool, 2016) Part 1** | | | | | | | |
| --- | --- | --- | --- | --- | --- | --- | --- |
| Study | **Abbas et al., 2021** | **Abbas et al., 2022** | **Adler et al., 2020** | **Barnett et al., 2020** | **Henson et al., 2020** | **Henson et al., 2021** | **Ranjan et al., 2022** |
| Introduction |  |  |  |  |  |  |  |
| 1. Were the aims/objectives of the study clear? | YES | YES | NO | YES | YES | YES | NO |
| Methods |  |  |  |  |  |  |  |
| 2. Was the study design appropriate for the stated aim(s)? | YES | YES | YES | YES | YES | YES | YES |
| 3. Was the sample size justified? | NO | NO | NO | NO | NO | NO | NO |
| 4. Was the target/reference population clearly defined? (Is it clear who the research was about?) | YES | YES | YES | YES | YES | YES | YES |
| 5. Was the sample frame taken from an appropriate population base so that it closely represented the target/reference population under investigation? | NO | YES | NO | NO | YES | YES | YES |
| 6. Was the selection process likely to select subjects/participants that were representative of the target/reference population under investigation? | NO | YES | YES | NO | YES | YES | YES |
| 7. Were measures undertaken to address and categorise non-responders? | NO | NO | NO | NO | YES | NO | NO |
| 8. Were the risk factor and outcome variables measured appropriate to the aims of the study? | NO | YES | NO | NO | NO | NO | NO |
| 9. Were the risk factor and outcome variables measured correctly using instruments/ measurements that had been trialled, piloted or published previously? | NO | YES | NO | NO | YES | NO | NO |
| 10. Is it clear what was used to determined statistical significance and/or precision estimates? (eg, p values, CIs) | YES | YES | YES | YES | NO | YES | YES |
| 11. Were the methods (including statistical methods) sufficiently described to enable them to be repeated? | YES | YES | YES | NO | NO | YES | YES |
| Results |  |  |  |  |  |  |  |
| 12. Were the basic data adequately described? | YES | YES | YES | YES | YES | YES | YES |
| 13. Does the response rate raise concerns about non-response bias? | NO | NO | NO | NO | NO | NO | NO |
| 14. If appropriate, was information about non-responders described? | NOT APPROPRIATE | NOT APPROPRIATE | NOT APPROPRIATE | NOT APPROPRIATE | NOT APPROPRIATE | NOT APPROPRIATE | NOT APPROPRIATE |
| 15. Were the results internally consistent? | YES | YES | YES | NO | NO | NO | NO |
| 16. Were the results for the analyses described in the methods, presented? | YES | YES | YES | YES | YES | YES | YES |

| **The Appraisal tool for Cross-Sectional Studies (AXIS tool, 2016) Part 2** | | | | | | | |
| --- | --- | --- | --- | --- | --- | --- | --- |
| Study | **Strauss et al., 2022** | **Wang et al., 2016** | **Busk et al., 2020** | **Ebner-Priemer et al., 2020** | **Jepsen et al., 2021** | **Tseng et al., 2022** | **Abbas et al., 2021** |
| Introduction |  |  |  |  |  |  |  |
| 1. Were the aims/objectives of the study clear? | YES | YES | YES | NO | YES | YES | YES |
| Methods |  |  |  |  |  |  |  |
| 2. Was the study design appropriate for the stated aim(s)? | YES | YES | YES | YES | YES | YES | YES |
| 3. Was the sample size justified? | NO | NO | NO | NO | NO | NO | NO |
| 4. Was the target/reference population clearly defined? (Is it clear who the research was about?) | YES | YES | YES | YES | YES | YES | YES |
| 5. Was the sample frame taken from an appropriate population base so that it closely represented the target/reference population under investigation? | YES | YES | NO | YES | YES | YES | YES |
| 6. Was the selection process likely to select subjects/participants that were representative of the target/reference population under investigation? | YES | YES | NO | NO | YES | YES | YES |
| 7. Were measures undertaken to address and categorise non-responders? | NO | YES | NO | NO | YES | NO | NO |
| 8. Were the risk factor and outcome variables measured appropriate to the aims of the study? | NO | YES | NO | NO | NO | NO | YES |
| 9. Were the risk factor and outcome variables measured correctly using instruments/ measurements that had been trialled, piloted or published previously? | NO | YES | NO | NO | NO | NO | YES |
| 10. Is it clear what was used to determined statistical significance and/or precision estimates? (eg, p values, CIs) | YES | YESY | YES | YES | YES | YES | YES |
| 11. Were the methods (including statistical methods) sufficiently described to enable them to be repeated? | YES | YES | YES | YES | YES | YES | YES |
| Results |  |  |  |  |  |  |  |
| 12. Were the basic data adequately described? | YES | YES | YES | YES | YES | YES | YES |
| 13. Does the response rate raise concerns about non-response bias? | NO | NO | NO | NO | NO | NO | NO |
| 14. If appropriate, was information about non-responders described? | NOT APPROPRIATE | NOT APPROPRIATE | NOT APPROPRIATE | NOT APPROPRIATE | NOT APPROPRIATE | NOT APPROPRIATE | NOT APPROPRIATE |
| 15. Were the results internally consistent? | YES | YES | YES | YES | YES | YES | YES |
| 16. Were the results for the analyses described in the methods, presented? | YES | YES | YES | YES | YES | YES | YES |

| **The Appraisal tool for Cross-Sectional Studies (AXIS tool, 2016) Part 3** | | | | | | | | |
| --- | --- | --- | --- | --- | --- | --- | --- | --- |
| Study | **Bai et al., 2021** | **Jacobson et al., 2020** | **Laiou et al., 2022** | **Pedrelli et al., 2020** | **Cho et al., 2020** | **Canzian et al., 2015** | **Chikersal et al., 2021** | **Cho et al., 2019** |
| Introduction |  |  |  |  |  |  |  |  |
| 1. Were the aims/objectives of the study clear? | YES | YES | YES | YES | YES | YES | YES | YES |
| Methods |  |  |  |  |  |  |  |  |
| 2. Was the study design appropriate for the stated aim(s)? | YES | YES | YES | YES | YES | YES | YES | YES |
| 3. Was the sample size justified? | NO | NO | NO | NO | NO | NO | NO | NO |
| 4. Was the target/reference population clearly defined? (Is it clear who the research was about?) | YES | NO | YES | YES | YES | NO | NO | YES |
| 5. Was the sample frame taken from an appropriate population base so that it closely represented the target/reference population under investigation? | YES | YES | YES | YES | YES | NO | NO | YES |
| 6. Was the selection process likely to select subjects/participants that were representative of the target/reference population under investigation? | NO | YES | NO | NO | YES | NO | NO | YES |
| 7. Were measures undertaken to address and categorise non-responders? | NO | YES | NO | YES | YES | NO | NO | YES |
| 8. Were the risk factor and outcome variables measured appropriate to the aims of the study? | YES | NO | NO | YES | NO | NO | NO | YES |
| 9. Were the risk factor and outcome variables measured correctly using instruments/ measurements that had been trialled, piloted or published previously? | YES | NO | NO | YES | NO | NO | NO | YES |
| 10. Is it clear what was used to determined statistical significance and/or precision estimates? (eg, p values, CIs) | YES | YES | YES | YES | YES | YES | YES | YES |
| 11. Were the methods (including statistical methods) sufficiently described to enable them to be repeated? | YES | YES | YES | YES | YES | YES | YES | YES |
| Results |  |  |  |  |  |  |  |  |
| 12. Were the basic data adequately described? | YES | YES | YES | YES | YES | YES | YES | YES |
| 13. Does the response rate raise concerns about non-response bias? | NO | NO | NO | NO | NO | NO | NO | NO |
| 14. If appropriate, was information about non-responders described? | NOT APPROPRIATE | NOT APPROPRIATE | NOT APPROPRIATE | YES | NOT APPROPRIATE | NOT APPROPRIATE | NOT APPROPRIATE | YES |
| 15. Were the results internally consistent? | YES | YES | YES | YES | YES | YES | YES | YES |
| 16. Were the results for the analyses described in the methods, presented? | YES | YES | YES | YES | YES | YES | YES | YES |

| **The Appraisal tool for Cross-Sectional Studies (AXIS tool, 2016) Part 4** | | | | | | | |
| --- | --- | --- | --- | --- | --- | --- | --- |
| Study | **Mehrotra et al., 2016** | **Wahle et al., 2016** | **Jacobson et al., 2020** | **Jacobson et al., 2021** | **Jacobson et al., 2022** | **Meyerhoff et al., 2021** | **Epstein et al., 2020** |
| Introduction |  |  |  |  |  |  |  |
| 1. Were the aims/objectives of the study clear? | YES | YES | YES | YES | YES | YES | YES |
| Methods |  |  |  |  |  |  |  |
| 2. Was the study design appropriate for the stated aim(s)? | YES | YES | YES | YES | YES | YES | YES |
| 3. Was the sample size justified? | NO | NO | NO | NO | NO | NO | NO |
| 4. Was the target/reference population clearly defined? (Is it clear who the research was about?) | NO | NO | NO | NO | NO | NO | YES |
| 5. Was the sample frame taken from an appropriate population base so that it closely represented the target/reference population under investigation? | NO | NO | NO | YES | NO | YES | YES |
| 6. Was the selection process likely to select subjects/participants that were representative of the target/reference population under investigation? | NO | NO | NO | YES | NO | NO | YES |
| 7. Were measures undertaken to address and categorise non-responders? | NO | NO | NO | NO | YES | YES | YES |
| 8. Were the risk factor and outcome variables measured appropriate to the aims of the study? | NO | YESY | YES | YES | YES | YES | YES |
| 9. Were the risk factor and outcome variables measured correctly using instruments/ measurements that had been trialled, piloted or published previously? | NO | YES | YES | YES | YES | YES | YES |
| 10. Is it clear what was used to determined statistical significance and/or precision estimates? (eg, p values, CIs) | YES | YES | YES | YES | YES | YES | YES |
| 11. Were the methods (including statistical methods) sufficiently described to enable them to be repeated? | YES | YES | YES | YES | YES | YES | YES |
| Results |  |  |  |  |  |  |  |
| 12. Were the basic data adequately described? | NO | YES | YES | YES | YES | YES | YES |
| 13. Does the response rate raise concerns about non-response bias? | NO | NO | NO | NO | NO | NO | NO |
| 14. If appropriate, was information about non-responders described? | NOT APPROPRIATE | NOT APPROPRIATE | NOT APPROPRIATE | NOT APPROPRIATE | NOT APPROPRIATE | YES | NOT APPROPRIATE |
| 15. Were the results internally consistent? | NO | YES | YES | YES | YES | YES | YES |
| 16. Were the results for the analyses described in the methods, presented? | NO | YES | YES | YES | YES | YES | YES |
